# Clonal Complex 17 Group B *Streptococcus* strains causing invasive disease in neonates and adults originate from the same genetic pool

**DOI:** 10.1038/srep20047

**Published:** 2016-02-04

**Authors:** Sarah Teatero, Erin Ramoutar, Allison McGeer, Aimin Li, Roberto G. Melano, Jessica Wasserscheid, Ken Dewar, Nahuel Fittipaldi

**Affiliations:** 1 Public Health Ontario, Toronto, ON, Canada; 2 Department of Laboratory Medicine and Pathobiology, Faculty of Medicine, University of Toronto, Toronto, ON, Canada; 3 Mount Sinai Hospital, Toronto, ON, Canada; 4 Department of Human Genetics, McGill University and Génome Québec Innovation Centre, Montreal, QC, Canada

## Abstract

A significant proportion of group B *Streptococcus* (GBS) neonatal disease, particularly late-onset disease, is associated with strains of serotype III, clonal complex (CC) 17. CC17 strains also cause invasive infections in adults. Little is known about the phylogenetic relationships of isolates recovered from neonatal and adult CC17 invasive infections. We performed whole-genome-based phylogenetic analysis of 93 temporally and geographically matched CC17 strains isolated from both neonatal and adult invasive infections in the metropolitan region of Toronto/Peel, Canada. We also mined the whole-genome data to reveal mobile genetic elements carrying antimicrobial resistance genes. We discovered that CC17 GBS strains causing neonatal and adult invasive disease are interspersed and cluster tightly in a phylogenetic tree, signifying that they are derived from the same genetic pool. We identified limited variation due to recombination in the core CC17 genome. We describe that loss of Pilus Island 1 and acquisition of different mobile genetic elements carrying determinants of antimicrobial resistance contribute to CC17 genetic diversity. Acquisition of some of these mobile genetic elements appears to correlate with clonal expansion of the strains that possess them. Our results provide a genome-wide portrait of the population structure and evolution of a major disease-causing clone of an opportunistic pathogen.

The opportunistic pathogen group B *Streptococcus* (GBS, also known as *Streptococcus agalactiae*) is one of the leading causes of neonatal bacterial infections worldwide, commonly resulting in pneumonia, septicemia, or meningitis^1^. GBS neonatal infections present as two distinct clinical syndromes: early-onset disease (EOD), characterized by onset of disease during the first 6 days of life; and late-onset disease (LOD), characterized by onset of symptoms from day 7 up to 3 months of age. The highest incidence of disease occurs during the first 3 months of life, but many cases have been recorded among infants aged >90 days, leading to the concept of ultra-LOD^2^. Decades of investigation have shown a remarkable association of serotype III, sequence type (ST) 17 GBS strains with neonatal disease, particularly with LOD^3^,^4^,^5^,^6^. Strains of this genetic background, and a few other closely related STs included in the clonal complex (CC) 17 have historically been designated as hypervirulent^6^,^7^,^8^. The virulence arsenal of ST17 strains has been deciphered in part^9^. Notably, strains of this genetic lineage possess an adhesin known as HvgA that confers an enhanced ability to invade the central nervous system of neonates^9^,^10^,^11^. Other surface adhesins such as Srr1, Srr2 and FbsC are thought to play important roles in GBS adaptation and host specificity^12^,^13^. In addition, three pilus islands (PI), PI-1, PI-2a, and PI-2b, which encode distinct pilus structures, have been identified in GBS and shown to mediate interactions with host cells^14^,^15^. Human isolates of GBS CC17 strains lack FbsC and Srr1 but possess Srr2 and both PI-1 and PI-2b^9^,^13^,^16^.

In addition to neonatal disease, GBS can cause disease in adults, particularly among the elderly and those that are immunocompromised^17^,^18^. Most cases of GBS disease in adults have been associated with serotype V strains, particularly those belonging to CC1^17^,^19^,^20^. We recently described the emergence of CC1 serotype IV among adult infections in Toronto, Canada^21^, and also noticed a substantial number of serotype III, CC17 strains among GBS adult invasive infections in the area^22^. Recently, Da Cunha *et al*. performed genomic analysis of a convenience sample of 71 temporally disparate CC17 isolates, recovered mainly in European and African countries, and described a relatively modest level of intra-CC17 phylogenetic diversity^23^. However, most isolates in the collection examined by these authors were from carriage (N = 24) or neonatal disease (N = 42) (5 strains were isolated from patients of unreported age)^23^. Thus, fundamental questions regarding the phylogenetic relationships between CC17 GBS strains causing disease in neonates and adults cannot be answered from these data. In particular, it is unclear if invasive strains causing disease in adults represent a distinct genetic subpopulation of CC17 strains or if, alternatively, they belong to the same population that causes invasive disease in neonates. Horizontal transfer of genetic material plays a significant role in GBS genome evolution^23^,^24^,^25^. Recently, it was hypothesized that expansion of GBS clones causing human disease was linked to acquisition of resistance to tetracycline^23^. It was also suggested that the expansion of CC1 GBS among adults may be associated with acquisition of resistance to macrolide and lincosamide antibiotics. Independent studies support this hypothesis^19^,^26^. However, it is unknown whether acquisition of antibiotic resistance is associated with age tropism in CC17 GBS.

Here, we sequenced the genomes of 93 temporally and geographically matched CC17 GBS strains isolated from both neonatal and adult invasive infections and performed whole-genome-based phylogenetic analysis to evaluate the hypothesis that CC17 strains causing disease in neonates and adults originate from the same genetic pool. We also investigated antimicrobial resistance gene content among CC17 strains. We report that CC17 strains associated with neonatal and adult disease are members of the same population, and provide evidence that acquisition of mobile genetic elements (MGEs) carrying antibiotic resistance genes, and loss of pilus islands contribute to variation among the CC17 GBS lineage.

## Results

### Strain collection, age groups and source of isolates

**Figure 1:**
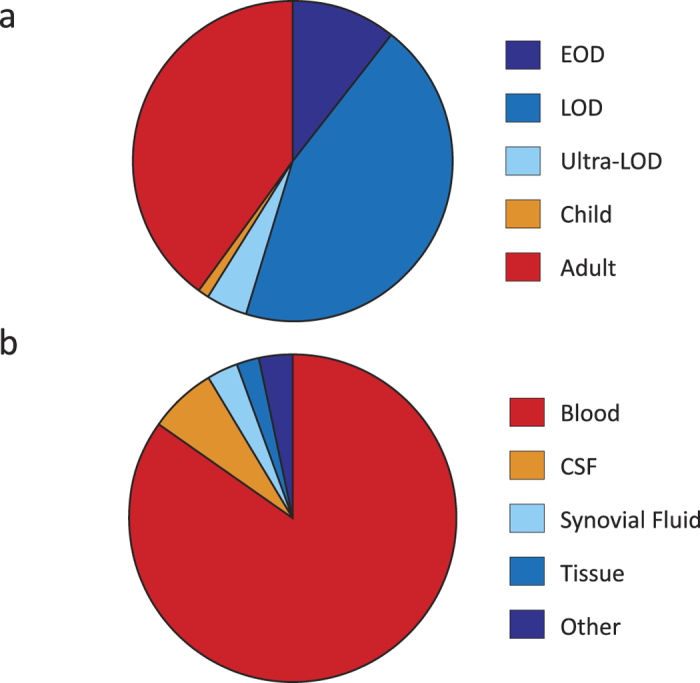
Age groups and source of isolation of CC17 GBS isolates. (**a**) Distribution of invasive GBS CC17 isolates by age. CC17 isolates were primarily isolated from neonatal disease: 11% EOD (early-onset disease, 0–6 day old, dark blue); 44% LOD (late-onset disease, 7–89 days old, blue); 4% Ultra-LOD (>90 days old, light blue). One isolate was collected from a child (1%, depicted in orange). 40% of the isolates were collected from adult patients (>18 years, red). **(b)** Distribution of CC17 isolates by source of isolation. Most invasive CC17 GBS were isolated from blood (79, 85%). Additionally, there were six isolates from cerebrospinal fluid (CSF), three isolates from synovial fluid, two from tissue, and three strains isolated from other normally sterile sites.

We assembled a population-based collection of 93 serotype III CC17 isolates (Table S1), representing 14% of the total burden of GBS invasive disease in the metropolitan area of Toronto/Peel, Canada, during the period of 2009 to 2012^22^. Most (N = 85, 91%) of the isolates were ST17. We also identified three ST148, two ST31, and one strain each of ST95, ST290, and ST484 among the serotype III collection. These other STs are all CC17 single locus variants of ST17. Ten strains (11%) were isolated from EOD cases (Fig. 1a). LOD isolates comprised 41 isolates (44%). The mean patient age for this group was 33 days old (range 7–87 days). Four strains (4%) were isolated from neonates >90 days (ultra-LOD, 4%); the mean age in this group was 105 days (range 95–117 days). One isolate was recovered from a child aged 10 years old. Thirty-seven isolates (40%) were collected from adults (average age 59 years; range 25.8–97.5 years). The isolates were recovered from blood (N=79, 85%), cerebrospinal fluid (N=6, 6%), synovial fluid (N=3, 3%), tissue (N=2, 2%), and pleural fluid (N=3, 3%) (Fig. 1b).

### Closure of a CC17 reference genome

**Figure 2:**
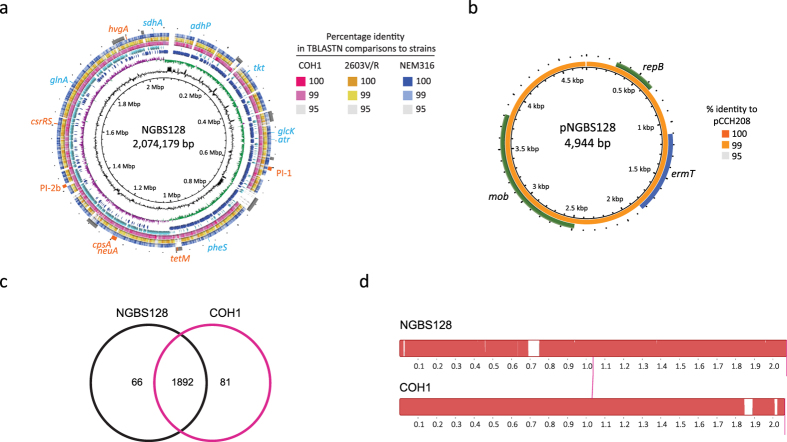
Whole-genome analysis of serotype III, ST17 GBS strain NGBS128. **(a**) Genome atlas of reference strain NGBS128. Genome scale in mega base pairs is given in the innermost circle (circle 1). GC content is displayed in circle 2 with values above average (outward directed) or below average (inward directed) indicated. Circle 3 shows GC skew, calculated as (G − C)/(G + C) and averaged over a moving window of 10,000 bp, showing excess G (green) and C (purple). Circle 4 shows CDS on the positive strand (dark blue) and circle 5 shows negative-strand encoded CDSs (light blue). TBLASTN comparisons of the reference NGBS128 genome to the complete genomes of strains COH1, 2603V/R and NEM316 are shown in pink, yellow, and blue on circles 6, 7 and 8, respectively. Genome landmarks are displayed in circle 9 and include MGEs (grey) and genes used in the GBS MLST scheme (blue). Other landmarks such as pilus islands, antimicrobial resistance genes, and capsule biosynthesis locus genes *cpsA-neuA* are shown in orange. **(b)** Atlas of *ermT*-containing plasmid pNGBS128. TBLASTN comparison of the 4,944 bp pNGBS128 plasmid to plasmid pCCH208 is shown in circle 1, in shades of orange. Annotated plasmid genes are shown in circle 2 (green and blue). **(c)** Venn diagram depicting orthologous gene cluster content in strains NGBS128 and the ST17 isolate COH1. Overlapping areas of circles represent common gene clusters. **(d)** Mauve alignment of the genomes of ST17 strains NGBS128 and COH1. Gaps in alignment correspond to mobile genetic elements.

A single ST17 GBS complete genome is available in GenBank (strain COH1, Accession number HG939456). However, this strain was isolated in the United States more than three decades ago^27^, and thus is temporally and geographically unrelated to our collection of CC17 organisms. To facilitate downstream whole-genome analysis, we decided to sequence to closure the genome of an arbitrarily chosen ST17 GBS strain from our collection. Strain NGBS128, isolated from a case of LOD in 2010 had a genome of 2,074,179 bp, encoding 1,981 CDSs, 21 rRNA, and 80 tRNA (Fig. 2a). The genome G + C content was 35.73%. We identified 8 major MGEs, including the *tetM*-containing integrative and conjugative element Tn5801. NGBS128 also had a 4,944 bp plasmid carrying the gene *ermT*. This plasmid (pNGBS128) was shown by BLASTN to be 99% similar to the previously described^28^ GBS plasmid pCCH208 (GenBank Accession KJ778678) (Fig. 2b). pNGBS128 was also highly similar to the GBS plasmid pGB2001 (GenBank Accession JF308630). We identified 1,892 common gene clusters between NGBS128 and COH1 (Fig. 2c). There was a high degree of synteny between genomes, and differences in gene content were mainly due to different MGE content (Fig. 2d). NGBS128 contains eight MGEs which combined comprise approximately 210 kbp, while COH1 also contains eight MGEs that combined comprise approximately 170 kbp. Despite the temporal and geographic differences between the strains, the NGBS128 core genome was >99.9% similar by BLASTN identity to strain COH1. We only identified 79 SNPs and 16 insertions/deletions between NGBS128 and COH1 in the concatenated sequence of the 1,892 common genes of these strains.

### Whole-genome, single-nucleotide polymorphism-based phylogeny of CC17 strains

To precisely define phylogenetic relationships between neonatal and adult CC17 strains we performed whole-genome SNP-based phylogenetic analysis. We identified a total of 2,442 polymorphisms among the 93 CC17 strains, or an average of 82 polymorphisms per strain (range: 9–250), relative to the core genome of reference strain NGBS128. We next evaluated recombination using BratNextGen. Results of this analysis identified two areas of recombination among 11 strains (Fig. S1 and Fig. S2). Although recombination is a common feature of GBS^24^, a recent report described low rates of recombination (3% of the genome) among CC17 strains^23^. Likely because our isolates are from the same geographical area and are temporally matched, our results showed an even lower rate of recombination (0.68% of the core genome of strain NGBS128), which involved an area of approximately 12 kbp in 9 strains, and an area of approximately 500 bp in 2 strains (Fig. S1 and Fig. S2). However, it is worth noting that the areas of the genome that we identified as having undergone recombination encode, among others, the two component system CiaHR (Fig. S2), which has been described as an important factor in GBS intracellular survival and invasive disease pathogenesis^29^.

**Figure 3:**
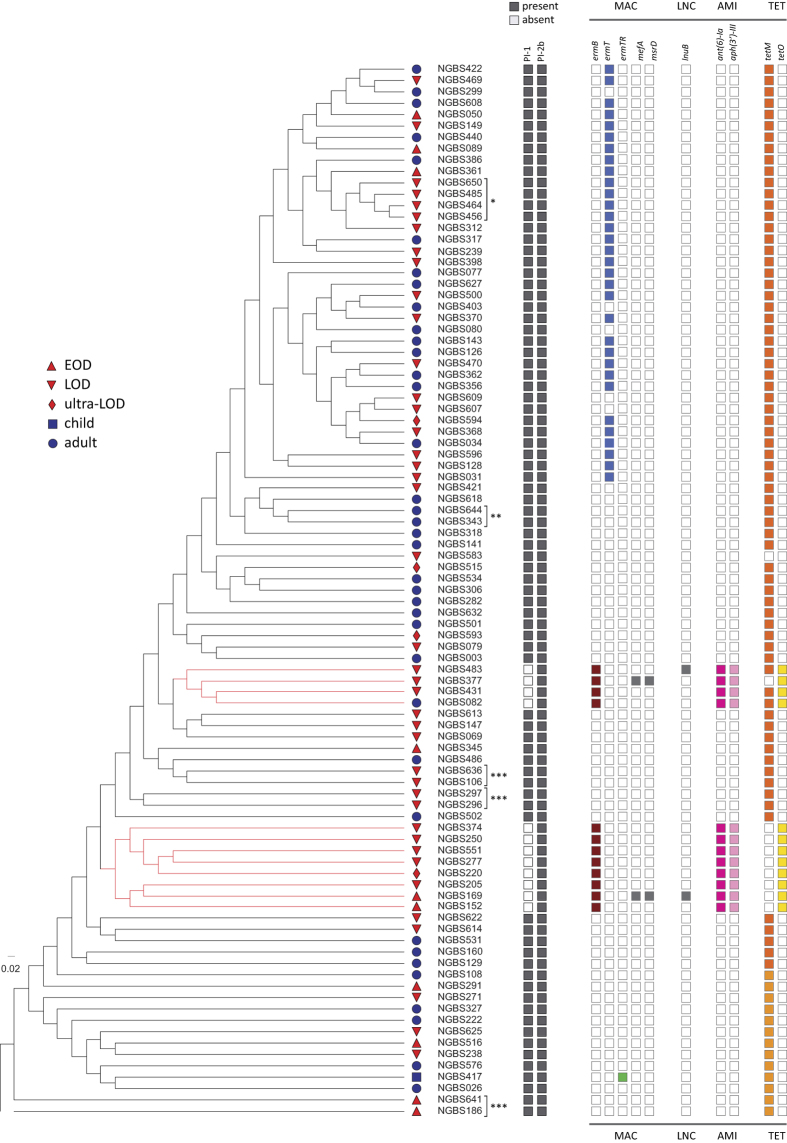
Phylogeny of the 93 CC17 isolates and distribution of pilus island and antibiotic resistance genes. The cladogram, constructed using 2,389 non-redundant single-nucleotide polymorphic loci identified among 93 serotype III CC17 isolates relative to the genome of ST17 reference strain NGBS128, shows the inferred phylogenetic relationships between CC17 strains causing invasive disease in Toronto, Canada. Age group to which the isolate belongs is shown by the following symbols; EOD (0–6 days, triangle), LOD (7–89 days, inverted triangle), ultra-LOD (90–120 days, diamond), child (120 days–18 years, square), adult (>18 years, circle). Neonatal infections (EOD, LOD and ultra-LOD) are colored in red. Strain names are listed; strains from a presumptive single patient are marked with asterisks (see also Table S1). None of the EOD/adult genetic pairs of strains were clinically linked. Presence of pilus island loci PI-1 and PI-2b is shown in grey. Presence of genes encoding resistance to macrolide (MAC), lincosamide (LNC), aminoglycoside (AMI), and tetracycline (TET) antibiotics is shown by colored boxes.

Once areas of recombination were removed from the analysis, the amount of genetic variation was very similar in the neonatal CC17 population (total of 1,390 SNPs, average of 79 SNPs per strain, relative to the core genome of the reference strain NGBS128) and the temporally and geographically matched adult CC17 population (total of 1,277 SNPs, average of 76 SNPs per strain, relative to the core genome of the reference strain) (see Tables S2 and S3). Thus, genetic variation in neonatal and adult CC17 GBS is virtually identical. To investigate the issue in more detail, we sought to determine if neonatal and adult strains clustered in identical or distinct branches of a neighbor-joining phylogenetic tree built using the 2,389 non-redundant SNPs identified among all strains in the collection relative to the core genome of the NGBS128 reference strain. The analysis revealed that, overall, adult and neonatal strains were interspersed and clustered tightly with one another in phylogenetic trees (Fig. 3 and Fig. S3). This finding effectively rules out the hypothesis that adult GBS isolates represent only one or a limited subset of CC17 lineages. Thus, we conclude that, as a population, CC17 strains causing adult and neonatal infections originate from the same genetic pool and appear to have closely similar evolutionary histories.

### Independent loss of Pilus Island 1 in some CC17 strains

CC17 strains have been shown to primarily possess PI-1 and PI-2b^30^,^31^, and this was the most common pilus profile found among our strains (Fig. 3). However, while all 93 isolates carried PI-2b, we discovered that the PI-1 locus was absent from the genomes of 12 strains forming two unrelated clades (Fig. 3, highlighted in red in the cladogram). Interestingly, among strains with PI-2b only, all but one were isolates recovered from neonatal infections (8 LOD, 1 ultra-LOD, and 2 EOD). The exception was one strain from an adult (31 years old, isolated in 2010). Loss of PI-1 is a common phenomenon in GBS^32^. However, among CC17, loss of PI-1 had only been described among bovine isolates^32^ until very recently, when a report from China showed that a majority (10 out 14) of CC17 isolates from human carriage were devoid of this locus^33^. The loss of PI-1 appears to have occurred independently twice in our strains, as revealed by phylogenetic analysis (Fig. 3). Interestingly, all strains of one of these clades (n = 8) had also undergone recombination at the two component system CiaHR locus (Fig. S1 and Fig. S2).

### Antibiotic resistance gene content among CC17 GBS

It has recently been suggested that acquisition of genetic determinants conferring tetracycline resistance has contributed to selection of GBS clones causing disease in humans^23^. Consistent with this hypothesis, we detected that 99% of the strains in our CC17 collection carried genes conferring tetracycline resistance. Most strains (91%) contained one of two *tetM* alleles (Fig. 3). Twelve strains (belonging to the abovementioned two clusters devoid of PI-1) possessed the *tetO* gene, and three of these strains had both *tetM* and *tetO*. Isolates possessing both *tetM* and *tetO* have likely independently acquired MGEs harboring these antibiotic resistance genes (see below). We identified a single strain that did not contain any tetracycline resistance gene.

An association between acquisition of resistance to macrolides and expansion of certain CC1 GBS clones causing disease in adults has recently been reported^23^,^26^. We identified that 48% of the CC17 strains in our collection possessed macrolide resistance determinants. Of note, 32 strains (34% of the collection) including the reference strain NGBS128 were found to possess a plasmid-encoded copy of *ermT* gene. This gene was recently described to occur on small, mobilizable, broad-host-range plasmids in both GBS and group A *Streptococcus*^34^. While our phylogenetic analysis is suggestive of ongoing expansion of the strains possessing *ermT*, we discovered that five strains had lost this plasmid in at least four independent events (Fig. 3). To investigate whether CC17 strains isolated in other geographies possess this plasmid, we downloaded from the NCBI’s Sequence Read Archive genome data for 70 CC17 isolates collected in several European and African countries, Australia and the United States^23^. We were unable to detect pNGBS128 in the short-read genome data of these strains. Phylogenetic analysis revealed that our CC17 strains carrying this *ermT*-containing plasmid clustered tightly in the phylogenetic tree (Fig. 3 and Fig. S4). Thus, strains containing pNGBS128 appear to be so far specific to Canada. We also discovered that all 12 strains that had lost PI-1 have acquired genes *ermB, tetO* and aminoglycoside resistance genes *ant(6*′*)-1a* and *aph(3*′*)-III*, likely in a single recombination event (see below). Interestingly, these 12 strains were for the most part (11/12, 92%) associated with neonatal disease. Among the abovementioned collection of GBS strains isolated elsewhere, only 2 of 70 CC17 isolates contained *ermB*, and both were from LOD cases^23^. In our collection, two strains possessing gene *ermB* also had macrolide resistance genes *mefA* and *msrD*. One of these strains also had the lincosamide resistance gene *lnuB*. One *ermB*-containing isolate also had gene *lnuB*, without other macrolide resistance genes. Finally, we identified one strain possessing a chromosomal copy of the *ermTR* gene (Fig. 3).

### Antibiotic resistance determinants are carried by distinct MGEs

**Figure 4:**
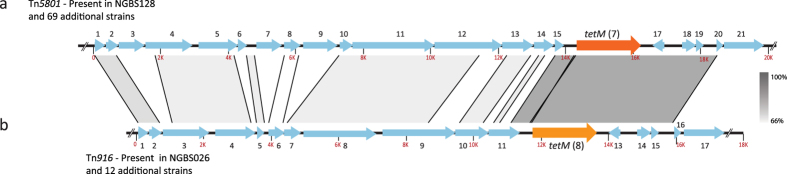
Two MGEs carry resistance to tetracycline encoded by gene *tetM*. **(a)** Tn*5801*, identified in 70 strains, including the reference strain NGBS128, carried *tetM* allele 7 (dark orange). **(b)** Tn*916* was identified in 13 strains and carried *tetM* allele 8 (light orange). BLAST identities are shown in shades of grey. All other genes are numbered and their predicted function is presented in Table S4.

**Figure 5:**
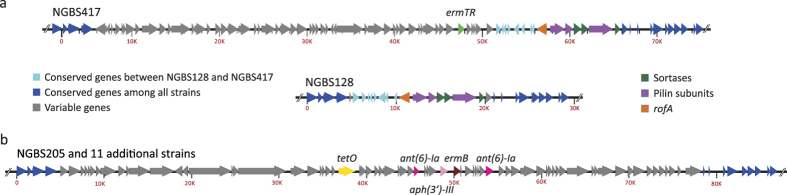
Pilus Island 1 is a hotspot for integration of MGEs carrying antimicrobial genes. **(a)** Chromosomal macrolide resistance was encoded by *ermTR* in a single strain. The top schematic shows *ermTR* gene (green) carried by a MGE which has inserted upstream of PI-1 in strain NGBS417. Depicted in the middle schematic is the region between nucleotides 589,950 and 619,400 (from the beginning of gene *aroA* to the end of gene *cylD*) in the genome of reference strain NGBS128. This region contains PI-1 genes, comprising the regulator *rofA* (orange), major and minor pilin subunits (purple), and three class C sortases (green). **(b)** Loss of PI-1 is associated with acquisition of an MGE carrying multiple resistance genes in several CC17 isolates. The bottom schematic shows that in 12 CC17 strains, PI-1 has been exchanged with a >70 kbp MGE carrying multiple antibiotic resistance genes. Conserved genes are indicated in blue, variable genes are indicated in grey. Antimicrobial resistance genes are indicated. Predicted function of genes is shown in Table S5.

MGEs make fundamental contributions to new habitat adaptation and the emergence of new lineages^25^,^35^. Strains in our collection have acquired and/or lost genes conferring resistance to several antibiotics. As mentioned above, gene *ermT* is carried on a plasmid. However, most other resistance genes were found to be chromosomally encoded. To investigate the issue in more detail, we sought to determine what MGEs carried these antibiotic genes. Our analysis identified two distinct MGEs carrying different *tetM* alleles among strains in our collection. The first, identified by BLASTN homology comparisons as Tn*5801* and carrying *tetM* allele 7, was identified in the reference strain NGBS128 and in 69 additional strains (Fig. 4a and Table S4). This ICE was found to be integrated at the 5′ end of the *guaA* gene, an insertion hotspot previously identified in GBS^24^. The amino acid sequence of this *tetM* allele was 100% similar to *tetM* sequences found in *Enterococcus faecalis*, *Staphylococcus aureus* and other GBS strains. The second ICE, identified by BLASTN homology comparisons as Tn*916* and carrying *tetM* allele 8, was found in strain NGBS026 and 12 additional strains (Fig. 4b and Table S4). These two *tetM* alleles are 92% similar by amino acid identity. We also identified an MGE in strain NGBS417, which carries macrolide resistance encoded by gene *ermTR*. This element was found immediately upstream of PI-1 (Fig. 5a and Table S5). Finally, we also identified that 12 strains possessed an MGE carrying *tetO, ermB, ant(6)-Ia, and aph(3*′*)-III*. When we examined the *de novo* assemblies of these strains, we discovered that the *ermB*-containing MGE had integrated at same genome location where PI-1 is expected to be found based on the genome sequence of reference strain NGBS128 (Fig. 5b and Table S5).

## Discussion

The phylogenetic relationships between CC17 strains causing neonatal and adult invasive disease is poorly known, in part because the GBS MLST scheme traditionally used for genotyping GBS strains does not permit sufficient discrimination between strains^36^. Specifically, it was not known whether disease in these two groups of age is caused by particular subsets of CC17 genotypes. Technological advances now allow us to address these issues at the whole-genome level in large populations of strains. Here, using whole-genome data for a temporally and geographically matched set of 93 CC17 strains, we unambiguously show that CC17 organisms causing EOD, LOD, ultra-LOD and adult disease originate from the same genetic pool and do not constitute separate genetic subsets. Instead, strains from neonates and adult disease cluster tightly and are interspersed in a phylogenetic tree. Another limitation of MLST is that it cannot identify extensive genomic variation in most cases. On the other hand, analysis of WGS data can easily detect recombination involving vast genomic regions. For example, we recently showed that a serotype V strain of ST297 (by MLST, a single-locus variant of CC1 founder ST1) has undergone recombination involving approximately 14% of its genome^22^. However, and despite the fact that large-scale recombination leading to capsular locus replacements in CC17 has been reported^22^,^37^, recombination analysis in our CC17 collection of invasive organisms did not uncover extensive core genome recombination. Recombination was detected mostly among ST17 strains, and only in one non-ST17 CC17 strain. While the non-ST17 STs included in our CC17 collection are single locus variants of ST17, the genomes of these non-ST17 strains only differed from the typical ST17 genome by, on average, 134 SNPs. Our results echo recent findings describing a relatively low rate of recombination among CC17 strains^23^.

Although core genome recombination among our strain collection was minimal, we identified that loss of genetic material resulting in strains that are devoid of PI-1 occurred on at least two independent occasions. Loss of pilus islands is common among non-CC17 GBS lineages^32^. It has also been reported previously among CC17 isolates of bovine origin^32^ but until very recently, all studied human CC17 isolates had been shown to possess this pilus island^38^,^39^. However, Lu *et al*. recently analyzed CC17 carriage isolates and found that most strains (10 out 14) were devoid of PI-1^33^. Since these authors did not sequence the genomes of these CC17 strains, we cannot relate our findings to their results. However, one hypothesis is that their and our CC17 isolates that lack PI-1 are members of a recently emerged clone that is expanding. Of note, all of our strains devoid of PI-1 have acquired a MGE element encoding resistance to tetracycline, macrolides and other antibiotics. Since the MGE was integrated at the same location where PI-1 is expected, we speculate that loss of PI-1 and acquisition of this MGE occurred simultaneously. Since PI-1 is a potential vaccine candidate^40^, loss or replacement of this locus may have implications for the protection offered by pilus subunit-based vaccines.

Our genomic analysis revealed that MGEs were also responsible for a relatively important level of non-core genome intra-CC17 variation. In a number of cases, these MGE carried genetic determinants of resistance against antimicrobial drugs. For example, we identified that most strains (92/93, 99%) possessed one of two *tetM* alleles, and/or the *tetO* gene, conferring resistance to tetracycline. These genes were found to be carried in at least three different MGEs. We also discovered that resistance to macrolides was encoded by genes carried on at least two different MGEs, and on one plasmid. It has been suggested that the acquisition of resistance to macrolides has led to expansion of CC1 GBS in adults^23^,^26^. While our data seems to suggest ongoing clonal expansion of CC17 strains possessing a plasmid carrying *ermT*, we did not find a clear association between adult CC17 disease and macrolide resistance.

In summary, we report here the population structure of CC17 GBS causing invasive infections in Toronto, Canada, and provide conclusive evidence showing that strains causing neonatal and adult disease originate from the same genetic pool. We also show low levels of intra-CC17 core genome recombination, but describe that PI-1 loss, and acquisition of both chromosomal and extrachromosomal MGEs encoding antimicrobial resistance contribute to diversity among this group of organisms. Our study highlights the usefulness of WGS data analysis of population-based strain samples to provide novel information about opportunistic pathogens causing human disease.

## Methods

### Isolate collection and growth conditions

Our collection comprised 93 previously described^22^ serotype III, CC17 GBS isolates recovered from patients with invasive diseases in the metropolitan region of Toronto/Peel, Canada (population under surveillance approx. 5,000,000), between January 2009 and October 2012 (listed in Table S1). For 5 patients, multiple isolates were included (collected either from more than one body site or on multiple dates). Therefore, our collection of 93 isolates represents 86 unique patients (Table S1). Strain COH1 was kindly provided by Dr. M. Segura (University of Montreal). GBS strains were grown in Todd Hewitt Broth supplemented with 0.2% yeast extract or on sheep blood agar plates at 37 °C with 5% CO_2_. DNA was prepared using the QIAamp DNA minikit (Qiagen, Toronto, ON, Canada) as per manufacturers’ recommendations for Gram-positive organisms.

### Whole-genome sequencing

Strain NGBS128 was arbitrarily chosen as the reference strain for genomic comparisons. Its genome was sequenced to closure using PacBio SMRT sequencing technology (Pacific Biosciences, Menlo Park, CA, USA), as previously described^26^. Briefly, two SMRT cells of sequence were generated, resulting in >70,000 reads exceeding 3 kb in length (average length of 5,374 bp, total coverage of 187×). To assess base-calling accuracy in the PacBio assembly, genomic libraries were generated using Nextera XT kits (Illumina, San Diego, CA, USA) and sequenced as paired-end (150 bp + 150 bp) with a MiSeq instrument (Illumina). The genome assembly was completely concordant with full length perfectly aligning Illumina short-reads. Since the PacBio protocol we used had a DNA size selection step with a cut-off of 20 kbp, we performed *de novo* assembly of the Illumina reads using the A5 pipeline^41^ to detect plasmid DNA. Generated contigs were aligned to the PacBio assemblies using progressiveMauve^42^. One contig of approximately 4.9 kbp did not align to the chromosomal assembly of strain NGBS128 and corresponded to plasmid DNA. We used primer walking and Sanger sequencing to circularize the plasmid (primers listed in Table S6). Genome annotation and identification of rRNA-encoding and tRNA-encoding regions were performed with Prokka^43^. MGEs of strain NGBS128 were defined with PHAST^44^ and by manual inspection of annotated sequences. Sequence data of strain NGBS128 and plasmid pNGBS128 were deposited in GenBank under accession number CP012480 and CP012742, respectively. The genomes of all remaining isolates, and the genome of strain COH1, were sequenced as paired-end reads (101 bp + 101 bp, or 150 bp + 150 bp) using HiSeq 2500 or MiSeq instruments (Illumina). The Sequence Read Archive (SRA) accession numbers for all isolates are provided in Table S1.

### MLST and Pilus Typing

MLST was determined directly from the whole-genome sequence reads using the read-mapping tool SRST2^45^. Pilus island content was assessed from *de novo* assemblies of short-read genome data generated using the A5 pipeline^41^, and by *in silico* alignment of genome sequence reads to pilus island genes using the MOSAIK aligner^46^. Results were confirmed using previously described PCR tests targeting GBS pilus islands^39^.

### Phylogenetic analysis and antimicrobial resistance determinants

Ortholog analysis was performed using PGAP^47^. Recombination analysis was performed using BRATNextGen^48^ run with 20 iterations and 100 replicates, with a p-value of 0.05 as the significance cut-off. Polymorphisms relative to the core genome (i.e. the approximately 1.9 Mbp area of the genome devoid of MGEs and areas of recombination) of strain NGBS128 were identified using the variant ascertainment algorithm VAAL^49^. Neighbor-joining phylogenetic trees (1,000 bootstrap replications) were constructed based on concatenated biallelic core SNPs using SplitsTree^50^ and TreeDyn^51^. Strain SS1^19^ was used to outroot the tree. SRST2 and a database of 1,913 genes were used to detect antibiotic resistant determinants among the CC17 GBS strains^45^. Contigs from *de novo* assemblies were ordered against the genome sequence of strain NGBS128 using progressiveMauve^42^, then concatenated using the sequence NNNNNCATTCCATTCATTAATTAATTAATGAATGAATGNNNNN, which introduces start and stop codons in all 6 reading frames, as a separator. MGEs that containing antibiotic resistance determinants were defined by manual inspection and BLAST of the *de novo* assembled contigs as described by Brochet *et al*.^24^. Genome visualizations were generated with BRIG^52^.

## Additional Information

**Accession codes:** Sequence data of NGBS128 and plasmid pNGBS128 was deposited in GenBank under accession numbers CP012480 and CP012742.

**How to cite this article**: Teatero, S. *et al*. Clonal Complex 17 Group B *Streptococcus* strains causing invasive disease in neonates and adults originate from the same genetic pool. *Sci. Rep*. **6**, 20047; doi: 10.1038/srep20047 (2016).

## Supplementary Material

Supplementary Information
