# Supplementary material for: Clonal Complex 17 Group B Streptococcus strains causing invasive disease in neonates and adults originate from the same genetic pool
Source: Sci Rep. 2016 Feb 4;6:20047. doi: 10.1038/srep20047 (PMC4740736; doi:10.1038/srep20047)
Supplement: Supplementary Information [file srep20047-s1.pdf]

**Supplementary Material for SREP-15-29607A. This file contains four supplementary figures (Figure S1-S4) and six supplementary tables (Table S1-S6).**

# Clonal Complex 17 Group B *Streptococcus* strains causing invasive disease in neonates and adults originate from the same genetic pool

**Sarah Teatero[1†], Erin Ramoutar[1†], Allison McGeer[2,3], Aimin Li[1], Roberto G. Melano[1,2,3], Jessica Wasserscheid[4], Ken Dewar[4], and Nahuel Fittipaldi[1,2]*.**

Public Health Ontario, Toronto, ON, Canada[1], Department of Laboratory Medicine and Pathobiology, Faculty of Medicine, University of Toronto, Toronto, ON, Canada[2], Mount Sinai Hospital, Toronto, ON, Canada[3], Department of Human Genetics, McGill University and Génome Québec Innovation Centre, Montreal, QC, Canada[4].

† These authors contributed equally to this work.

* Author for correspondence: Nahuel Fittipaldi, PhD. Public Health Ontario, Toronto Laboratories, 661 University Ave., Suite 19-099, Toronto, Ontario M5G 1M1, Canada. Phone: (647) 792-3429. E-mail: nahuel.fittipaldi@oahpp.ca.

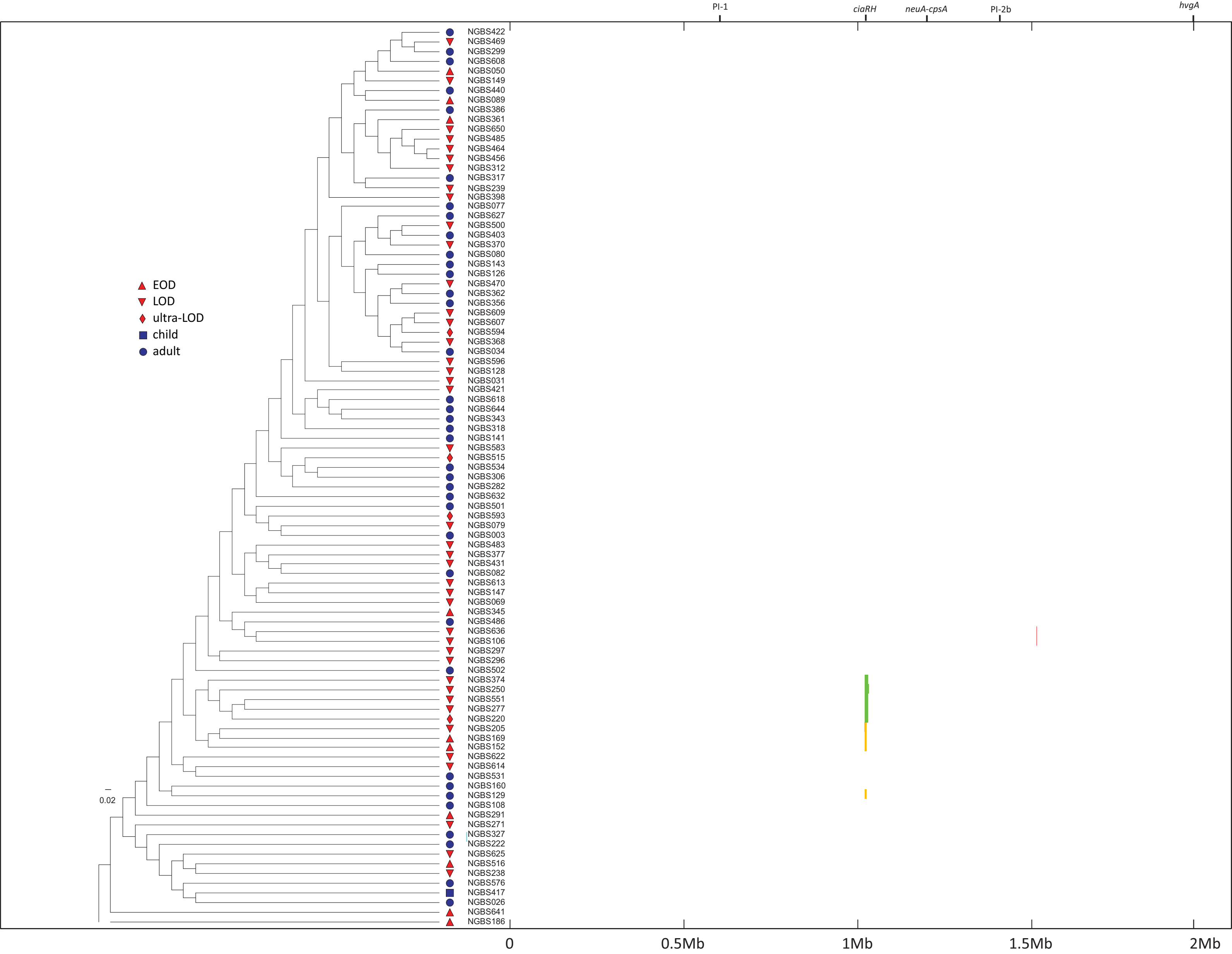


**Figure S1. Recombination in CC17 GBS isolates.** The cladogram depicted in Fig. 3 is shown on left-hand side. Genome size of the reference strain (NGBS128) is marked in 0.5Mbp increments along the bottom. Recombination is shown in the right-hand panel as vertical coloured bars. The colouring of the bars at a specific genomic location reflects the clustering of the recombination events into groups, and is unrelated to other bars at distant genomic locations.

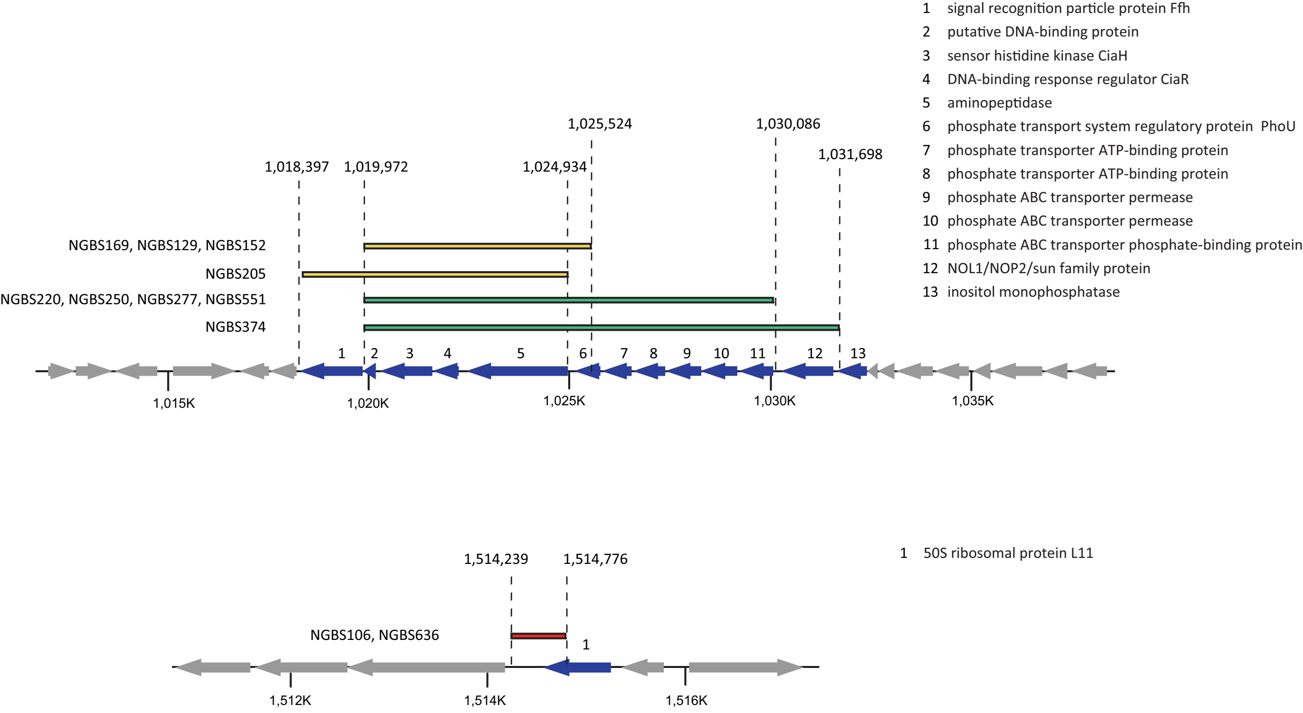


**Figure S2. Genes found within recombined areas of CC17 GBS isolates.** Genes found in the two areas of recombination described in Fig. S1 are shown as numbered blue arrows. Numbers correspond to gene names shown on the right. Surrounding genes not affected by recombination are coloured grey. Areas of recombination are shown by horizontal coloured bars and the start and end positions (relative to the genome of strain NGBS128) are marked by dashed lines.

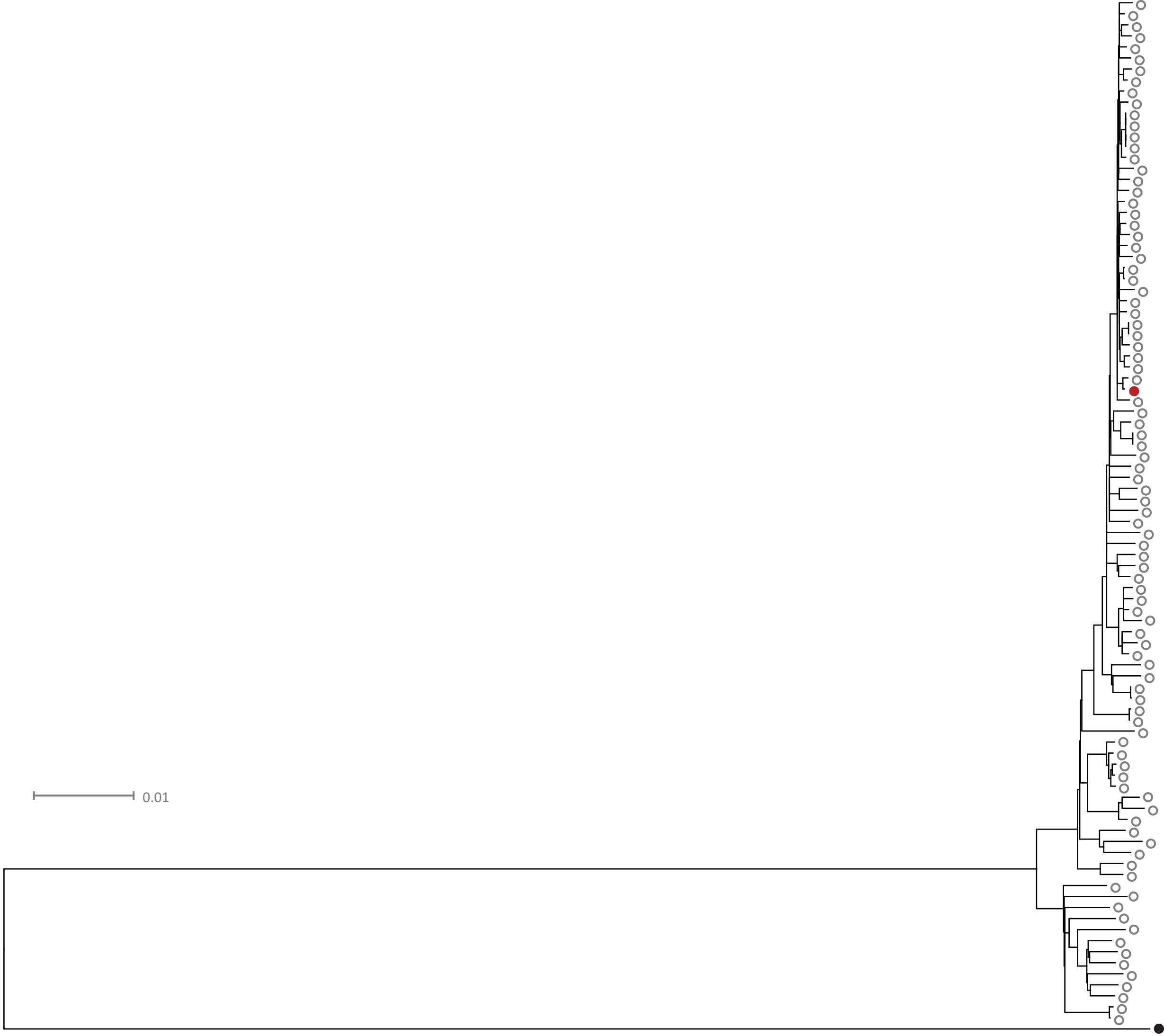


**Figure S3. Rooted phylogenetic tree of CC17 strains.** Phylogeny based on 13,876 non-redundant single-nucleotide polymorphic loci identified among the genomes of 93 serotype III CC17 isolates plus serotype V ST1 strain SS1 relative to the ST17 reference strain NGBS128. CC17 isolates represented by open grey circles. Reference NGBS128 is marked in red. Strain SS1 is shown as black filled circle.

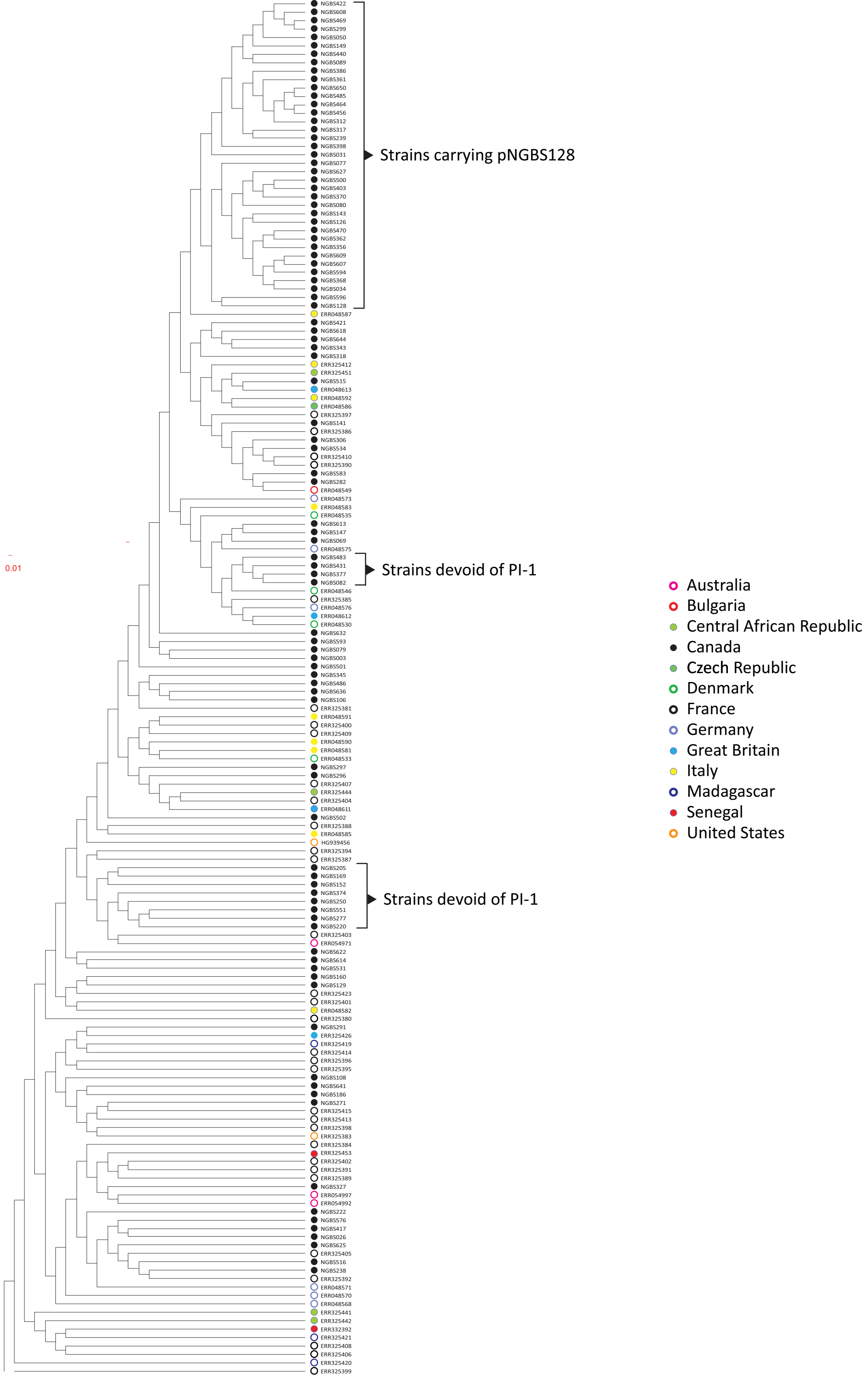


Figure S4. Inferred phylogenetic relationships between 93 CC17 GBS strains causing invasive disease in Canada (this study) and a previously described (DaCunha *et al*. Nature Communications 5, Article number: 4544 doi:10.1038/ncomms5544) strain collection comprising 71 ST17 carriage and neonatal disease isolates recovered in different countries. A cladogram was constructed using the concatenated sequences of 5,324 non-redundant single-nucleotide polymorphic loci relative to the genome of reference strain NGBS128. Country of origin is indicated by the differently coloured circles.

**Table S1. CC17 GBS isolates used in this study.**

| Strain Name | Isolation Year | Age Group | Sequence Type | Pilus Content | Source [1] | SRA Accession Number |
|---|---|---|---|---|---|---|
| NGBS003 | 2009 | Adult | 17 | PI-1+2b | Blood | SAMN04093966 |
| NGBS026 | 2009 | Adult | 17 | PI-1+2b | Blood | SAMN04093967 |
| NGBS031 | 2009 | LOD | 17 | PI-1+2b | Blood | SAMN04093968 |
| NGBS034 | 2010 | Adult | 17 | PI-1+2b | Blood | SAMN04093969 |
| NGBS050 | 2010 | EOD | 17 | PI-1+2b | Blood | SAMN04093970 |
| NGBS069 | 2010 | LOD | 17 | PI-1+2b | CSF | SAMN04093971 |
| NGBS077 | 2010 | Adult | 17 | PI-1+2b | Blood | SAMN04093972 |
| NGBS079 | 2010 | LOD | 17 | PI-1+2b | Blood | SAMN04093973 |
| NGBS080 | 2010 | Adult | 17 | PI-1+2b | Blood | SAMN04093974 |
| NGBS082 | 2010 | Adult | 17 | PI-2b | Blood | SAMN04093975 |
| NGBS089 | 2010 | EOD | 17 | PI-1+2b | Blood | SAMN04093976 |
| NGBS106 [2] | 2010 | LOD | 17 | PI-1+2b | CSF | SAMN04093977 |
| NGBS108 | 2010 | Adult | 17 | PI-1+2b | Blood | SAMN04093978 |
| NGBS126 | 2010 | Adult | 17 | PI-1+2b | Other | SAMN04093979 |
| NGBS128 | 2010 | LOD | 17 | PI-1+2b | Blood | SAMN04007140 |
| NGBS129 | 2010 | Adult | 31 | PI-1+2b | Blood | SAMN04093980 |
| NGBS141 | 2010 | Adult | 17 | PI-1+2b | Blood | SAMN04093981 |
| NGBS143 | 2010 | Adult | 17 | PI-1+2b | Other | SAMN04093982 |
| NGBS147 | 2010 | LOD | 17 | PI-1+2b | Blood | SAMN04093983 |
| NGBS149 | 2010 | LOD | 17 | PI-1+2b | Blood | SAMN04093984 |
| NGBS152 | 2010 | EOD | 17 | PI-2b | Blood | SAMN04093985 |
| NGBS160 | 2010 | Adult | 31 | PI-1+2b | Tissue | SAMN04093986 |
| NGBS169 | 2010 | EOD | 17 | PI-2b | Blood | SAMN04093987 |
| NGBS186 [3] | 2010 | EOD | 17 | PI-1+2b | Blood | SAMN04093988 |
| NGBS205 | 2011 | LOD | 17 | PI-2b | Blood | SAMN04093989 |
| NGBS220 | 2011 | Ultra LOD | 17 | PI-2b | Blood | SAMN04093990 |
| NGBS222 | 2010 | Adult | 17 | PI-1+2b | Blood | SAMN04093991 |
| NGBS238 | 2011 | LOD | 17 | PI-1+2b | Blood | SAMN04093992 |
| NGBS239 | 2011 | LOD | 17 | PI-1+2b | Blood | SAMN04093993 |
| NGBS250 | 2011 | LOD | 17 | PI-2b | CSF | SAMN04093994 |
| NGBS271 | 2011 | LOD | 17 | PI-1+2b | CSF | SAMN04093995 |
| NGBS277 | 2010 | LOD | 17 | PI-2b | Blood | SAMN04093996 |
| NGBS282 | 2010 | Adult | 17 | PI-1+2b | Blood | SAMN04093997 |
| NGBS291 | 2010 | EOD | 17 | PI-1+2b | Blood | SAMN04093998 |
| NGBS296 [4] | 2010 | LOD | 17 | PI-1+2b | Blood | SAMN04093999 |
| NGBS297 [4] | 2010 | LOD | 17 | PI-1+2b | Blood | SAMN04094000 |
| NGBS299 | 2011 | Adult | 17 | PI-1+2b | Blood | SAMN04094001 |
| NGBS306 | 2011 | Adult | 17 | PI-1+2b | Blood | SAMN04094002 |

| | | | | | | |
|---|---|---|---|---|---|---|
| NGBS312 | 2011 | LOD | 17 | PI-1+2b | Blood | SAMN04094003 |
| NGBS317 | 2011 | Adult | 17 | PI-1+2b | Blood | SAMN04094004 |
| NGBS318 | 2011 | Adult | 290 | PI-1+2b | Blood | SAMN04094005 |
| NGBS327 | 2011 | Adult | 484 | PI-1+2b | Blood | SAMN04094006 |
| NGBS343 [5] | 2011 | Adult | 17 | PI-1+2b | Blood | SAMN04094007 |
| NGBS345 | 2011 | EOD | 17 | PI-1+2b | Blood | SAMN04094008 |
| NGBS356 | 2011 | Adult | 17 | PI-1+2b | Blood | SAMN04094009 |
| NGBS361 | 2011 | EOD | 17 | PI-1+2b | Blood | SAMN04094010 |
| NGBS362 | 2011 | Adult | 17 | PI-1+2b | Blood | SAMN04094011 |
| NGBS368 | 2011 | LOD | 17 | PI-1+2b | Blood | SAMN04094012 |
| NGBS370 | 2011 | LOD | 17 | PI-1+2b | Blood | SAMN04094013 |
| NGBS374 | 2011 | LOD | 17 | PI-2b | Blood | SAMN04094014 |
| NGBS377 | 2011 | LOD | 17 | PI-2b | Blood | SAMN04094015 |
| NGBS386 | 2011 | Adult | 17 | PI-1+2b | Synovial Fluid | SAMN04094016 |
| NGBS398 | 2011 | LOD | 17 | PI-1+2b | Blood | SAMN04094017 |
| NGBS403 | 2011 | Adult | 17 | PI-1+2b | Blood | SAMN04094018 |
| NGBS417 | 2011 | Child | 17 | PI-1+2b | Synovial Fluid | SAMN04094019 |
| NGBS421 | 2011 | LOD | 17 | PI-1+2b | Blood | SAMN04094020 |
| NGBS422 | 2011 | Adult | 17 | PI-1+2b | Blood | SAMN04094021 |
| NGBS431 | 2011 | LOD | 17 | PI-2b | Blood | SAMN04094022 |
| NGBS440 | 2011 | Adult | 17 | PI-1+2b | Blood | SAMN04094023 |
| NGBS456 [6] | 2011 | LOD | 17 | PI-1+2b | Blood | SAMN04094024 |
| NGBS464 [6] | 2011 | LOD | 17 | PI-1+2b | Blood | SAMN04094025 |
| NGBS469 | 2011 | LOD | 17 | PI-1+2b | Blood | SAMN04094026 |
| NGBS470 | 2011 | LOD | 17 | PI-1+2b | Blood | SAMN04094027 |
| NGBS483 | 2011 | LOD | 17 | PI-2b | Blood | SAMN04094028 |
| NGBS485 [6] | 2011 | LOD | 17 | PI-1+2b | Blood | SAMN04094029 |
| NGBS486 | 2011 | Adult | 17 | PI-1+2b | Blood | SAMN04094030 |
| NGBS500 | 2012 | LOD | 17 | PI-1+2b | Blood | SAMN04094031 |
| NGBS501 | 2012 | Adult | 17 | PI-1+2b | Blood | SAMN04094032 |
| NGBS502 | 2012 | Adult | 95 | PI-1+2b | Blood | SAMN04094033 |
| NGBS515 | 2012 | Ultra LOD | 17 | PI-1+2b | Blood | SAMN04094034 |
| NGBS516 | 2012 | EOD | 17 | PI-1+2b | Blood | SAMN04094035 |
| NGBS531 | 2012 | Adult | 148 | PI-1+2b | Blood | SAMN04094036 |
| NGBS534 | 2012 | Adult | 17 | PI-1+2b | Blood | SAMN04094037 |
| NGBS551 | 2012 | LOD | 17 | PI-2b | Blood | SAMN04094038 |
| NGBS576 | 2012 | Adult | 17 | PI-1+2b | Blood | SAMN04094039 |
| NGBS583 | 2012 | LOD | 17 | PI-1+2b | Blood | SAMN04094040 |
| NGBS593 | 2012 | Ultra LOD | 17 | PI-1+2b | Blood | SAMN04094041 |

| | | | | | | |
|---|---|---|---|---|---|---|
| NGBS594 | 2012 | Ultra LOD | 17 | PI-1+2b | Blood | SAMN04094042 |
| NGBS596 | 2012 | LOD | 17 | PI-1+2b | Blood | SAMN04094043 |
| NGBS607 | 2012 | LOD | 17 | PI-1+2b | Blood | SAMN04094044 |
| NGBS608 | 2012 | Adult | 17 | PI-1+2b | Other | SAMN04094045 |
| NGBS609 | 2012 | LOD | 17 | PI-1+2b | Blood | SAMN04094046 |
| NGBS613 | 2012 | LOD | 17 | PI-1+2b | Blood | SAMN04094047 |
| NGBS614 | 2012 | LOD | 148 | PI-1+2b | CSF | SAMN04094048 |
| NGBS618 | 2012 | Adult | 17 | PI-1+2b | Blood | SAMN04094049 |
| NGBS622 | 2012 | LOD | 148 | PI-1+2b | Blood | SAMN04094050 |
| NGBS625 | 2012 | LOD | 17 | PI-1+2b | Blood | SAMN04094051 |
| NGBS627 | 2012 | Adult | 17 | PI-1+2b | Blood | SAMN04094052 |
| NGBS632 | 2012 | Adult | 17 | PI-1+2b | Tissue | SAMN04094053 |
| NGBS636 [2] | 2010 | LOD | 17 | PI-1+2b | Blood | SAMN04094054 |
| NGBS641 [3] | 2010 | EOD | 17 | PI-1+2b | Blood | SAMN04094055 |
| NGBS644 [5] | 2011 | Adult | 17 | PI-1+2b | Synovial Fluid | SAMN04094056 |
| NGBS650 [6] | 2011 | LOD | 17 | PI-1+2b | CSF | SAMN04094057 |

[1] CSF, cerebrospinal fluid.

[2, 3, 4, 5, 6] Isolates indicated by the same superscript number were isolated from a single patient.

**Table S2. Pairwise SNP comparison of adult CC17 strains.**

| | NGBS128 | NGBS003 | NGBS026 | NGBS034 | NGBS077 | NGBS080 | NGBS082 | NGBS108 | NGBS126 | NGBS129 | NGBS141 | NGBS143 | NGBS160 | NGBS222 | NGBS282 | NGBS299 | NGBS306 | NGBS317 | NGBS318 | NGBS327 | NGBS343 | NGBS356 | NGBS362 | NGBS386 | NGBS403 | NGBS422 | NGBS440 | NGBS486 | NGBS501 | NGBS502 | NGBS531 | NGBS534 | NGBS576 | NGBS608 | NGBS618 | NGBS627 | NGBS632 |
|---|---|---|---|---|---|---|---|---|---|---|---|---|---|---|---|---|---|---|---|---|---|---|---|---|---|---|---|---|---|---|---|---|---|---|---|---|---|
| NGBS128 | | | | | | | | | | | | | | | | | | | | | | | | | | | | | | | | | | | | | |
| NGBS003 | 63 | | | | | | | | | | | | | | | | | | | | | | | | | | | | | | | | | | | | |
| NGBS026 | 228 | 243 | | | | | | | | | | | | | | | | | | | | | | | | | | | | | | | | | | | |
| NGBS034 | 27 | 70 | 235 | | | | | | | | | | | | | | | | | | | | | | | | | | | | | | | | | | |
| NGBS077 | 18 | 63 | 228 | 23 | | | | | | | | | | | | | | | | | | | | | | | | | | | | | | | | | |
| NGBS080 | 29 | 74 | 239 | 34 | 29 | | | | | | | | | | | | | | | | | | | | | | | | | | | | | | | | |
| NGBS082 | 60 | 75 | 240 | 67 | 60 | 71 | | | | | | | | | | | | | | | | | | | | | | | | | | | | | | | |
| NGBS108 | 218 | 233 | 124 | 225 | 218 | 229 | 230 | | | | | | | | | | | | | | | | | | | | | | | | | | | | | | |
| NGBS126 | 18 | 63 | 228 | 21 | 16 | 25 | 60 | 218 | | | | | | | | | | | | | | | | | | | | | | | | | | | | | |
| NGBS129 | 126 | 141 | 222 | 133 | 126 | 137 | 138 | 214 | 126 | | | | | | | | | | | | | | | | | | | | | | | | | | | | |
| NGBS141 | 49 | 72 | 237 | 56 | 49 | 60 | 69 | 227 | 49 | 135 | | | | | | | | | | | | | | | | | | | | | | | | | | | |
| NGBS143 | 18 | 63 | 228 | 21 | 16 | 27 | 60 | 218 | 2 | 126 | 49 | | | | | | | | | | | | | | | | | | | | | | | | | | |
| NGBS160 | 126 | 141 | 228 | 133 | 126 | 137 | 138 | 218 | 126 | 62 | 135 | 126 | | | | | | | | | | | | | | | | | | | | | | | | | |
| NGBS222 | 240 | 255 | 112 | 247 | 240 | 251 | 252 | 136 | 240 | 234 | 249 | 240 | 240 | | | | | | | | | | | | | | | | | | | | | | | | |
| NGBS282 | 47 | 70 | 235 | 54 | 47 | 58 | 67 | 225 | 47 | 133 | 56 | 47 | 133 | 247 | | | | | | | | | | | | | | | | | | | | | | | |
| NGBS299 | 30 | 73 | 238 | 37 | 30 | 41 | 70 | 228 | 30 | 136 | 59 | 30 | 136 | 250 | 57 | | | | | | | | | | | | | | | | | | | | | | |
| NGBS306 | 57 | 80 | 245 | 64 | 57 | 68 | 77 | 235 | 57 | 143 | 66 | 57 | 143 | 257 | 64 | 67 | | | | | | | | | | | | | | | | | | | | | |
| NGBS317 | 27 | 70 | 235 | 34 | 27 | 38 | 67 | 225 | 27 | 133 | 56 | 27 | 133 | 247 | 54 | 33 | 64 | | | | | | | | | | | | | | | | | | | | |
| NGBS318 | 54 | 77 | 242 | 61 | 54 | 65 | 74 | 232 | 54 | 140 | 63 | 54 | 140 | 254 | 61 | 64 | 71 | 61 | | | | | | | | | | | | | | | | | | | |
| NGBS327 | 230 | 245 | 124 | 237 | 230 | 241 | 242 | 128 | 230 | 224 | 239 | 230 | 230 | 136 | 237 | 240 | 247 | 237 | 244 | | | | | | | | | | | | | | | | | | |
| NGBS343 | 51 | 76 | 241 | 58 | 51 | 62 | 73 | 231 | 51 | 139 | 62 | 51 | 139 | 253 | 60 | 61 | 70 | 58 | 63 | 243 | | | | | | | | | | | | | | | | | |
| NGBS356 | 24 | 67 | 232 | 25 | 20 | 31 | 64 | 222 | 18 | 130 | 53 | 18 | 130 | 244 | 51 | 34 | 61 | 31 | 58 | 234 | 55 | | | | | | | | | | | | | | | | |
| NGBS362 | 23 | 66 | 231 | 24 | 19 | 30 | 63 | 221 | 17 | 129 | 52 | 17 | 129 | 243 | 50 | 33 | 60 | 30 | 57 | 233 | 54 | 21 | | | | | | | | | | | | | | | |

| | | | | | | | | | | | | | | | | | | | | | | | | | | | | | | | | | | | | | |
|---|---|---|---|---|---|---|---|---|---|---|---|---|---|---|---|---|---|---|---|---|---|---|---|---|---|---|---|---|---|---|---|---|---|---|---|---|---|
| NGBS386 | 19 | 62 | 227 | 26 | 19 | 30 | 59 | 217 | 19 | 125 | 48 | 19 | 125 | 239 | 46 | 25 | 56 | 22 | 53 | 229 | 50 | 23 | 22 | | | | | | | | | | | | | | |
| NGBS403 | 22 | 65 | 230 | 23 | 18 | 27 | 62 | 220 | 16 | 128 | 51 | 16 | 128 | 242 | 49 | 32 | 59 | 29 | 56 | 232 | 53 | 20 | 19 | 21 | | | | | | | | | | | | | |
| NGBS422 | 31 | 74 | 239 | 38 | 31 | 42 | 71 | 229 | 31 | 137 | 60 | 31 | 137 | 251 | 58 | 35 | 68 | 34 | 65 | 241 | 62 | 35 | 34 | 26 | 33 | | | | | | | | | | | | |
| NGBS440 | 30 | 73 | 238 | 37 | 30 | 41 | 70 | 228 | 30 | 136 | 59 | 30 | 136 | 250 | 57 | 34 | 67 | 33 | 64 | 240 | 61 | 34 | 33 | 25 | 32 | 35 | | | | | | | | | | | |
| NGBS486 | 83 | 98 | 251 | 90 | 83 | 94 | 95 | 241 | 83 | 149 | 92 | 83 | 149 | 263 | 90 | 93 | 100 | 90 | 97 | 253 | 96 | 87 | 86 | 82 | 85 | 94 | 93 | | | | | | | | | | |
| NGBS501 | 61 | 76 | 241 | 68 | 61 | 72 | 73 | 231 | 61 | 139 | 70 | 61 | 139 | 253 | 68 | 71 | 78 | 68 | 75 | 243 | 74 | 65 | 64 | 60 | 63 | 72 | 71 | 96 | | | | | | | | | |
| NGBS502 | 130 | 145 | 242 | 137 | 130 | 141 | 142 | 232 | 130 | 138 | 139 | 130 | 140 | 254 | 137 | 140 | 147 | 137 | 144 | 244 | 143 | 134 | 133 | 129 | 132 | 141 | 140 | 153 | 143 | | | | | | | | |
| NGBS531 | 148 | 163 | 252 | 155 | 148 | 159 | 160 | 242 | 148 | 150 | 157 | 148 | 152 | 264 | 155 | 158 | 165 | 155 | 162 | 252 | 161 | 152 | 151 | 147 | 150 | 159 | 158 | 171 | 161 | 162 | | | | | | | |
| NGBS534 | 57 | 80 | 245 | 64 | 57 | 68 | 77 | 235 | 57 | 143 | 66 | 57 | 143 | 257 | 64 | 67 | 48 | 64 | 71 | 247 | 70 | 61 | 60 | 56 | 59 | 68 | 67 | 100 | 78 | 147 | 165 | | | | | | |
| NGBS576 | 239 | 254 | 85 | 246 | 239 | 250 | 251 | 135 | 239 | 233 | 248 | 239 | 239 | 123 | 246 | 249 | 256 | 246 | 253 | 135 | 252 | 243 | 242 | 238 | 241 | 250 | 249 | 262 | 252 | 253 | 263 | 256 | | | | | |
| NGBS608 | 20 | 63 | 228 | 27 | 20 | 31 | 60 | 218 | 20 | 126 | 49 | 20 | 126 | 240 | 47 | 24 | 57 | 23 | 54 | 230 | 51 | 24 | 23 | 15 | 22 | 25 | 24 | 83 | 61 | 130 | 148 | 57 | 239 | | | | |
| NGBS618 | 48 | 71 | 236 | 55 | 48 | 59 | 68 | 226 | 48 | 134 | 57 | 48 | 134 | 248 | 55 | 58 | 65 | 55 | 58 | 238 | 29 | 52 | 51 | 47 | 50 | 59 | 58 | 91 | 69 | 138 | 156 | 65 | 247 | 48 | | | |
| NGBS627 | 21 | 66 | 231 | 24 | 17 | 28 | 63 | 221 | 17 | 129 | 52 | 17 | 129 | 243 | 50 | 33 | 60 | 30 | 57 | 233 | 54 | 21 | 20 | 22 | 17 | 34 | 33 | 86 | 64 | 133 | 151 | 60 | 242 | 23 | 51 | | |
| NGBS632 | 70 | 85 | 250 | 75 | 70 | 81 | 82 | 240 | 70 | 148 | 79 | 70 | 148 | 262 | 77 | 80 | 87 | 77 | 84 | 252 | 83 | 74 | 73 | 69 | 72 | 81 | 80 | 105 | 83 | 152 | 170 | 87 | 261 | 70 | 78 | 73 | |
| NGBS644 | 51 | 76 | 241 | 58 | 51 | 62 | 73 | 231 | 51 | 139 | 62 | 51 | 139 | 253 | 60 | 61 | 70 | 58 | 63 | 243 | 0 | 55 | 54 | 50 | 53 | 62 | 61 | 96 | 74 | 143 | 161 | 70 | 252 | 51 | 29 | 54 | 83 |

11

**Table S3. Pairwise SNP comparison in Neonatal CC17 Strains**

| | NGBS128 | NGBS031 | NGBS050 | NGBS069 | NGBS079 | NGBS089 | NGBS106 | NGBS128 | NGBS147 | NGBS149 | NGBS152 | NGBS169 | NGBS186 | NGBS205 | NGBS220 | NGBS238 | NGBS239 | NGBS250 | NGBS271 | NGBS277 | NGBS291 | NGBS296 | NGBS297 | NGBS312 | NGBS345 | NGBS361 | NGBS368 | NGBS370 | NGBS374 | NGBS377 | NGBS398 | NGBS421 | NGBS431 | NGBS456 | NGBS464 | NGBS469 | NGBS470 | NGBS483 | NGBS485 | NGBS500 | NGBS515 | NGBS516 | NGBS551 | NGBS583 | NGBS593 | NGBS594 | NGBS596 | NGBS607 | NGBS609 | NGBS613 | NGBS614 | NGBS622 | NGBS625 | NGBS636 | NGBS641 | NGBS650 |
|---|---|---|---|---|---|---|---|---|---|---|---|---|---|---|---|---|---|---|---|---|---|---|---|---|---|---|---|---|---|---|---|---|---|---|---|---|---|---|---|---|---|---|---|---|---|---|---|---|---|---|---|---|---|---|---|---|
| NGBS128 | | | | | | | | | | | | | | | | | | | | | | | | | | | | | | | | | | | | | | | | | | | | | | | | | | | | | | | | |
| NGBS031 | 27 | | | | | | | | | | | | | | | | | | | | | | | | | | | | | | | | | | | | | | | | | | | | | | | | | | | | | | | |
| NGBS050 | 23 | 30 | | | | | | | | | | | | | | | | | | | | | | | | | | | | | | | | | | | | | | | | | | | | | | | | | | | | | | |
| NGBS069 | 51 | 60 | 56 | | | | | | | | | | | | | | | | | | | | | | | | | | | | | | | | | | | | | | | | | | | | | | | | | | | | | |
| NGBS079 | 56 | 63 | 59 | 61 | | | | | | | | | | | | | | | | | | | | | | | | | | | | | | | | | | | | | | | | | | | | | | | | | | | | |
| NGBS089 | 22 | 31 | 21 | 55 | 60 | | | | | | | | | | | | | | | | | | | | | | | | | | | | | | | | | | | | | | | | | | | | | | | | | | | |
| NGBS106 | 68 | 75 | 71 | 73 | 76 | 72 | | | | | | | | | | | | | | | | | | | | | | | | | | | | | | | | | | | | | | | | | | | | | | | | | | |
| NGBS128 | 0 | 27 | 23 | 51 | 56 | 22 | 68 | | | | | | | | | | | | | | | | | | | | | | | | | | | | | | | | | | | | | | | | | | | | | | | | | |
| NGBS147 | 57 | 64 | 60 | 20 | 65 | 61 | 77 | 57 | | | | | | | | | | | | | | | | | | | | | | | | | | | | | | | | | | | | | | | | | | | | | | | | |
| NGBS149 | 29 | 36 | 26 | 62 | 65 | 27 | 77 | 29 | 66 | | | | | | | | | | | | | | | | | | | | | | | | | | | | | | | | | | | | | | | | | | | | | | | |
| NGBS152 | 122 | 131 | 127 | 127 | 132 | 126 | 132 | 122 | 133 | 133 | | | | | | | | | | | | | | | | | | | | | | | | | | | | | | | | | | | | | | | | | | | | | | |
| NGBS169 | 148 | 155 | 151 | 153 | 156 | 152 | 156 | 148 | 157 | 157 | 46 | | | | | | | | | | | | | | | | | | | | | | | | | | | | | | | | | | | | | | | | | | | | | |
| NGBS186 | 219 | 228 | 223 | 226 | 229 | 225 | 229 | 219 | 230 | 230 | 229 | 249 | | | | | | | | | | | | | | | | | | | | | | | | | | | | | | | | | | | | | | | | | | | | |
| NGBS205 | 141 | 148 | 144 | 146 | 149 | 145 | 149 | 141 | 150 | 150 | 39 | 53 | 242 | | | | | | | | | | | | | | | | | | | | | | | | | | | | | | | | | | | | | | | | | | | |
| NGBS220 | 105 | 114 | 110 | 114 | 117 | 111 | 117 | 105 | 118 | 116 | 97 | 117 | 210 | 110 | | | | | | | | | | | | | | | | | | | | | | | | | | | | | | | | | | | | | | | | | | |
| NGBS238 | 228 | 235 | 231 | 233 | 236 | 232 | 236 | 228 | 237 | 236 | 234 | 254 | 133 | 247 | 217 | | | | | | | | | | | | | | | | | | | | | | | | | | | | | | | | | | | | | | | | | |
| NGBS239 | 33 | 40 | 32 | 66 | 69 | 33 | 81 | 33 | 70 | 38 | 137 | 161 | 234 | 154 | 120 | 241 | | | | | | | | | | | | | | | | | | | | | | | | | | | | | | | | | | | | | | | | |
| NGBS250 | 101 | 110 | 106 | 108 | 113 | 105 | 113 | 101 | 114 | 112 | 91 | 117 | 212 | 110 | 16 | 217 | 116 | | | | | | | | | | | | | | | | | | | | | | | | | | | | | | | | | | | | | | | |
| NGBS271 | 218 | 227 | 223 | 223 | 228 | 222 | 228 | 218 | 229 | 229 | 224 | 250 | 127 | 243 | 213 | 128 | 233 | 207 | | | | | | | | | | | | | | | | | | | | | | | | | | | | | | | | | | | | | | |
| NGBS277 | 109 | 116 | 112 | 114 | 117 | 113 | 117 | 109 | 118 | 118 | 95 | 115 | 210 | 108 | 12 | 215 | 122 | 14 | 211 | | | | | | | | | | | | | | | | | | | | | | | | | | | | | | | | | | | | | |
| NGBS291 | 246 | 253 | 249 | 249 | 254 | 250 | 254 | 246 | 255 | 255 | 252 | 272 | 153 | 265 | 235 | 154 | 259 | 235 | 150 | 233 | | | | | | | | | | | | | | | | | | | | | | | | | | | | | | | | | | | | |
| NGBS296 | 92 | 99 | 95 | 97 | 100 | 94 | 100 | 92 | 101 | 101 | 136 | 156 | 231 | 149 | 119 | 236 | 105 | 117 | 232 | 117 | 254 | | | | | | | | | | | | | | | | | | | | | | | | | | | | | | | | | | | |
| NGBS297 | 90 | 97 | 93 | 95 | 98 | 92 | 98 | 90 | 99 | 99 | 134 | 154 | 229 | 147 | 117 | 234 | 103 | 115 | 230 | 115 | 252 | 2 | | | | | | | | | | | | | | | | | | | | | | | | | | | | | | | | | | |
| NGBS312 | 21 | 28 | 20 | 54 | 57 | 21 | 69 | 21 | 58 | 26 | 125 | 149 | 222 | 142 | 108 | 229 | 30 | 104 | 221 | 110 | 247 | 93 | 91 | | | | | | | | | | | | | | | | | | | | | | | | | | | | | | | | | |

NGBS345 79 88 84 84 89 83 63 79 90 90 143 169 242 162 130 249 94 124 239 130 267 113 111 82

NGBS361 25 32 24 58 61 25 73 25 62 30 129 153 226 146 112 233 34 108 225 114 251 97 95 18 86

NGBS368 27 34 30 60 63 31 75 27 64 36 131 155 228 148 114 235 40 110 227 116 253 99 97 28 88 32

NGBS370 24 31 27 57 60 28 72 24 61 33 128 152 225 145 111 232 37 107 224 113 250 96 94 25 85 29 25

NGBS374 106 113 109 111 114 110 114 106 115 115 92 112 207 105 19 212 119 21 208 17 230 114 112 107 127 111 113 110

NGBS377 72 79 75 45 80 76 92 72 49 81 148 172 245 165 133 252 85 129 244 133 270 116 114 73 105 77 79 76 130

NGBS398 25 32 26 58 61 27 73 25 62 32 129 153 226 146 112 233 36 108 225 114 251 97 95 24 86 28 32 29 111 77

NGBS421 51 58 54 64 67 55 79 51 68 60 135 159 232 152 118 239 64 116 231 120 257 103 101 52 92 56 58 55 117 83 56

NGBS431 54 61 57 27 62 58 74 54 31 63 130 154 227 147 115 234 67 111 226 115 252 98 96 55 87 59 61 58 112 32 59 65

NGBS456 21 28 20 54 57 21 69 21 58 26 125 149 222 142 108 229 30 104 221 110 247 93 91 12 82 18 28 25 107 73 24 52 55

NGBS464 21 28 20 54 57 21 69 21 58 26 125 149 222 142 108 229 30 104 221 110 247 93 91 12 82 18 28 25 107 73 24 52 55 0

NGBS469 25 32 22 58 61 23 73 25 62 28 129 153 226 146 112 233 34 108 225 114 251 97 95 22 86 26 32 29 111 77 28 56 59 22 22

NGBS470 34 41 37 67 70 38 82 34 71 43 138 162 235 155 121 242 47 117 234 123 260 106 104 35 95 39 35 32 120 86 39 65 68 35 35 39

NGBS483 60 67 63 33 68 64 80 60 37 69 136 160 233 153 121 240 73 117 232 121 258 104 102 61 93 65 67 64 118 38 65 71 20 61 61 65 74

NGBS485 21 28 20 54 57 21 69 21 58 26 125 149 222 142 108 229 30 104 221 110 247 93 91 12 82 18 28 25 107 73 24 52 55 0 0 22 35 61

NGBS500 27 34 28 60 63 31 75 27 64 36 131 155 226 148 114 235 40 110 227 116 253 99 97 28 88 32 28 23 113 79 32 58 61 28 28 32 35 67 28

NGBS515 59 66 62 72 75 63 87 59 76 68 143 167 240 160 126 247 72 124 239 128 265 111 109 60 100 64 66 63 125 91 64 70 73 60 60 64 73 79 60 66

NGBS516 229 236 232 234 237 233 237 229 238 237 235 259 138 252 222 73 242 218 129 220 159 241 239 230 248 234 236 233 217 253 234 240 235 230 230 234 243 241 230 236 248

NGBS551 107 114 110 112 115 111 115 107 116 116 93 113 208 106 10 213 120 12 209 8 231 115 113 108 128 112 114 111 15 131 112 118 113 108 108 112 121 119 108 114 126 218

NGBS583 46 53 49 59 62 50 74 46 63 55 130 154 227 147 113 234 59 111 226 115 252 98 96 47 87 51 53 50 112 78 51 57 60 47 47 51 60 66 47 53 65 235 113

NGBS593 63 70 66 68 43 67 83 63 72 72 139 163 236 156 124 243 76 120 235 124 261 107 105 64 96 68 70 67 121 87 68 74 69 64 64 68 77 75 64 70 82 244 122 69

NGBS594 27 34 30 60 63 31 75 27 64 36 131 155 228 148 114 235 40 110 227 116 253 99 97 28 88 32 26 25 113 79 32 58 61 28 28 32 35 67 28 28 66 236 114 53 70

NGBS596 9 32 28 56 61 27 73 9 62 34 127 153 224 146 110 233 38 106 223 114 251 97 95 26 84 30 32 29 111 77 30 56 59 26 26 30 39 65 26 32 64 234 112 51 68 32

NGBS607 26 33 29 59 62 30 74 26 63 35 130 154 227 147 113 234 39 109 226 115 252 98 96 27 87 31 25 24 112 78 31 57 60 27 27 31 34 66 27 27 65 235 113 52 69 19 31

NGBS609 26 33 29 59 62 30 74 26 63 35 130 154 227 147 113 234 39 109 226 115 252 98 96 27 87 31 25 24 112 78 31 57 60 27 27 31 34 66 27 27 65 235 113 52 69 19 31 0

NGBS613 66 73 69 29 74 70 86 66 33 75 142 166 239 159 127 246 79 123 238 127 264 110 108 67 99 71 73 70 124 58 71 77 40 67 67 71 80 46 67 73 85 247 125 72 81 73 71 72 72

NGBS614 130 139 135 135 140 134 140 130 141 141 136 162 235 155 125 240 145 119 230 123 258 144 142 133 151 137 139 136 120 156 137 143 138 133 133 137 146 144 133 139 151 241 121 138 147 139 135 138 138 150

NGBS622 125 132 128 132 135 129 135 125 136 134 133 153 226 146 116 231 138 116 227 114 249 135 133 126 148 130 132 129 111 151 130 138 133 126 126 130 139 139 126 132 146 236 112 133 142 132 130 131 131 145 79

NGBS625 222 229 225 227 230 226 230 222 231 230 228 250 129 243 213 70 235 211 122 211 150 232 230 223 243 227 229 226 208 246 227 233 228 223 223 227 236 234 223 229 241 73 209 228 237 229 227 228 228 240 234 227

| | | | | | | | | | | | | | | | | | | | | | | | | | | | | | | | | | | | | | | | | | | | | | | | | | | | | | | | |
|---|---|---|---|---|---|---|---|---|---|---|---|---|---|---|---|---|---|---|---|---|---|---|---|---|---|---|---|---|---|---|---|---|---|---|---|---|---|---|---|---|---|---|---|---|---|---|---|---|---|---|---|---|---|---|---|
| NGBS636 | 69 | 76 | 72 | 74 | 77 | 73 | 1 | 69 | 78 | 78 | 133 | 157 | 230 | 150 | 118 | 237 | 82 | 114 | 229 | 118 | 255 | 101 | 99 | 70 | 64 | 74 | 76 | 73 | 115 | 93 | 74 | 80 | 75 | 70 | 70 | 74 | 83 | 81 | 70 | 76 | 88 | 238 | 116 | 75 | 84 | 76 | 74 | 75 | 75 | 87 | 141 | 136 | 231 | | |
| NGBS641 | 226 | 233 | 228 | 231 | 234 | 230 | 234 | 226 | 235 | 235 | 232 | 252 | 7 | 245 | 215 | 130 | 239 | 215 | 124 | 213 | 152 | 234 | 232 | 227 | 247 | 231 | 233 | 230 | 210 | 250 | 231 | 237 | 232 | 227 | 227 | 231 | 240 | 238 | 227 | 231 | 245 | 135 | 211 | 232 | 241 | 233 | 231 | 232 | 232 | 244 | 238 | 229 | 126 | 235 | |
| NGBS650 | 21 | 28 | 20 | 54 | 57 | 21 | 69 | 21 | 58 | 26 | 125 | 149 | 222 | 142 | 108 | 229 | 30 | 104 | 221 | 110 | 247 | 93 | 91 | 12 | 82 | 18 | 28 | 25 | 107 | 73 | 24 | 52 | 55 | 0 | 0 | 22 | 35 | 61 | 0 | 28 | 60 | 230 | 108 | 47 | 64 | 28 | 26 | 27 | 27 | 67 | 133 | 126 | 223 | 70 | 227 |

Table S4. Predicted function of genes found in MGEs depicted in Figure 4.

| Genetic Element | Gene Number (Fig. 4) | Putative function | Encoded Protein size (aa) | Top BLASTp hit description | GenBank Accession Number | % Identity (/aa) |
|---|---|---|---|---|---|---|
| Tn*5801* | 1 | Tn916 hypothetical protein | 104 | MULTISPECIES: hypothetical protein [Bacilli] | WP_000421 280.1 | 100% (/104) |
| | 2 | Tn916 hypothetical protein | 125 | MULTISPECIES: hypothetical protein [Bacilli] | WP_001234 189.1 | 100% (/125) |
| | 3 | Hypothetical protein | 257 | MULTISPECIES: hypothetical protein [Bacilli] | WP_000185 760.1 | 100% (/257) |
| | 4 | DNA translocase FtsK | 467 | MULTISPECIES: cell division protein FtsK [Bacilli] | WP_002320 760.1 | 100% (/467) |
| | 5 | Transcriptional regulator, Tn916 | 394 | MULTISPECIES: Cro/Cl family transcriptional regulator [Bacilli] | WP_000426 691.1 | 100% (/394) |
| | 6 | Tn916 hypothetical protein | 90 | MULTISPECIES: hypothetical protein [Bacilli] | WP_000055 367.1 | 100% (/90) |
| | 7 | Tn916 hypothetical protein | 253 | Hypothetical protein [*Streptococcus agalactiae*] | WP_047199 803.1 | 100% (/253) |
| | 8 | Tn916 ORF16 ATP/GTP-binding protein | 166 | MULTISPECIES: antirestriction protein ArdA [Bacilli] | WP_000421 245.1 | 99% (/166) |
| | 9 | Hypothetical protein | 341 | Hypothetical protein HMPREF9524_00211 [*Enterococcus faecium* TX0133a01] | EFR69551.1 | 100% (/341) |
| | 10 | Hypothetical protein | 131 | Tn*916* ORF17-like protein lmo1107 [*Streptococcus agalactiae* COH1] | EAO75913.1 | 100% (/131) |
| | 11 | ATP/GTP-binding protein | 817 | MULTISPECIES: ATP/GTP-binding protein [Bacilli] | WP_000941 001.1 | 100% (/817) |
| | 12 | Transmembrane amino acid transporter protein | 675 | MULTISPECIES: membrane protein [Bacilli] | WP_000192 390.1 | 100% (/675) |
| | 13 | Cell wall hydrolase, lytic transglycolase | 309 | Peptidase P60 [*Streptococcus agalactiae*] | WP_000768 375.1 | 100% (/309) |
| | 14 | Tn*916* ORF13 protein | 198 | Tn*916* ORF13 protein [*Streptococcus agalactiae*] | CFR23594.1 | 100% (/198) |

| | | | | | | |
|---|---|---|---|---|---|---|
| | 15 | Tn*916* ORF13 protein | 91 | Tn*916* ORF13 protein [*Streptococcus agalactiae*] | CFQ21202.1 | 100% (/91) |
| | 16 | Tetracycline resistance protein TetM | 639 | MULTISPECIES: tetracycline resistance protein TetM [Firmicutes] | WP_000691737.1 | 100% (/639) |
| | 17 | Transcriptional regulator, Tn916 | 117 | MULTISPECIES: DNA-binding protein [Bacilli] | WP_001227349.1 | 100% (/117) |
| | 18 | Tn916 hypothetical protein | 140 | conserved hypothetical protein [*Enterococcus faecalis* T8] | EEU24686.1 | 100% (/140) |
| | 19 | Polyribonucleotide nucleotidyltransferase | 76 | MULTISPECIES: hypothetical protein [Bacilli] | WP_000845144.1 | 100% (/76) |
| | 20 | Hypothetical protein | 66 | Conserved hypothetical protein *[Streptococcus agalactiae* COH1] | EAO75937.1 | 100% (/66) |
| | 21 | Site-specific recombinase, phage integrase family | 397 | MULTISPECIES: integrase [Bacilli] | WP_000237798.1 | 100% (/397) |
| **Tn*916*** | 1 | Tn*916* hypothetical protein | 104 | MULTISPECIES: hypothetical protein [Bacteria] | WP_000420682.1 | 100% (/104) |
| | 2 | Tn*916* hypothetical protein | 127 | MULTISPECIES: hypothetical protein [Bacteria] | WP_000985015.1 | 100% (/127) |
| | 3 | DNA translocase FtsK | 461 | MULTISPECIES: cell division protein FtsK [Bacteria] | WP_000813488.1 | 100% (/461) |
| | 4 | Cro/CI family transcriptional regulator | 401 | Cro/Cl family transcriptional regulator [*Streptococcus pseudopneumoniae*] | WP_049519213.1 | 100% (/401) |
| | 5 | Tn*916* hypothetical protein | 73 | MULTISPECIES: conjugal transfer protein [Bacteria] | WP_001009056.1 | 100% (/73) |
| | 6 | Tn*916* hypothetical protein | 165 | MULTISPECIES: antirestriction protein ArdA [Bacteria] | WP_000342539.1 | 100% (/165) |
| | 7 | Tn*916* hypothetical protein | 168 | MULTISPECIES: membrane protein [Bacteria] | WP_000506270.1 | 100% (/168) |
| | 8 | Tn*916* ORF16 ATP/GTP-binding protein | 815 | MULTISPECIES: ATP/GTP-binding protein [Bacteria] | WP_000331160.1 | 100% (/815) |
| | 9 | Tn*916* ORF15 signal peptide containing protein | 725 | MULTISPECIES: membrane protein [Bacteria] | WP_000804748.1 | 100% (/725) |
| | 10 | Tn*916*, NLP/P60 family protein | 333 | Peptidase P60 [*Streptococcus agalactiae*] | WP_029141002.1 | 99% (/333) |

| | | | | | |
|---|---|---|---|---|---|
| 11 | Tn*916* ORF13 protein | 311 | hypothetical protein SII_0646 [*Streptococcus intermedius* C270] | AGU77833.1 | 100% (/311) |
| 12 | Tetracycline resistance protein TetM | 639 | tetracycline resistance ribosomal protection protein [*Streptococcus agalactiae*] | WP_000691753.1 | 100% (/639) |
| 13 | Tn*916*, transcriptional regulator | 117 | MULTISPECIES: DNA-binding protein [Bacteria] | WP_001227347.1 | 100% (/117) |
| 14 | Tn*916* hypothetical protein | 140 | MULTISPECIES: DNA-binding protein [Bacteria] | WP_000804885.1 | 100% (/140) |
| 15 | Tn*916* hypothetical protein | 76 | MULTISPECIES: hypothetical protein [Bacteria] | WP_000857133.1 | 100% (/76) |
| 16 | Tn*916*, excisionase | 67 | MULTISPECIES: excisionase [Bacteria] | WP_000814511.1 | 100% (/67) |
| 17 | Tn*916*, transposase | 405 | MULTISPECIES: transposase [Bacteria] | WP_001291561.1 | 100% (/405) |

Table S5. Predicted function of genes found in the strains depicted in Figure 5.

| | NGBS128 | NGBS417 | NGBS205 |
|---|---|---|---|
| 1 | AroA | AroA | AroA |
| 2 | AroK | AroK | AroK |
| 3 | Psr protein | Psr protein | Psr protein |
| 4 | RNA methyltransferase | RNA methyltransferase | RNA methyltransferase |
| 5 | group II intron-interrupted relaxase ltrB | Double zinc ribbon | Hypothetical protein |
| 6 | AphA | PlcR family transcriptional regulator | Replication initiator protein A |
| 7 | Cytoplasmic protein | Hypothetical protein | Hypothetical protein |
| 8 | TetR family transcriptional regulator | Positive control sigma-like factor | C-5 cytosine-specific DNA methylase |
| 9 | TetR family transcriptional regulator | Hypothetical protein | Hypothetical protein |
| 10 | Cell wall surface anchor family protein, truncation | Phage protein | Arsenate reductase |
| 11 | IS5 family transposase orfB | Phage protein | Hypothetical protein |
| 12 | Transposase | Phage protein | protease |
| 13 | Chaperone protein HslO | Hypothetical protein | Hypothetical protein |
| 14 | AraC family transcriptional regulator | Prophage LambdaSa04, DAN polymerase | Type IV secretory pathway, VirD4 component |
| 15 | Cell wall surface anchor family protein | Phage regulatory protein, Rha family | membrane protein |
| 16 | Cell wall surface anchor family protein | Hypothetical protein | Orf23 |
| 17 | Sortase family protein | Hypothetical protein | Orf25 |
| 18 | Sortase | Prophage LambdaSa04, DNA primase | Orf26 |
| 19 | Cell wall surface anchor family protein | Phage protein | Orf28 |
| 20 | Sortase family protein | Prophage LambdaSa04, SNF2 family helicase | AbiGII |
| 21 | Cna B domain-containing protein | Hypothetical protein | AbiGI |
| 22 | Orf28 | Phage endonuclease | Agglutinin receptor |
| 23 | Hypothetical protein | Phage terminase, small subunit | Calcium-binding protein |
| 24 | Hypothetical protein | S-adenosylmethionine synthetase | Hypothetical protein |
| 25 | Lipoprotein | adenine-specific methyltrasferase | SNF2 family protein |
| 26 | ABC transporter ATP-binding protein | DNA methylase | group II intron reverse transcriptase/maturase |
| 27 | ABC transporter ATP-binding protein | Hypothetical protein | SNF2 family protein |
| 28 | Membrane protein | Hypothetical protein | Hypothetical protein |
| 29 | ABC transporter permeae | Hypothetical protein | Membrane protein |

| | | | |
|---|---|---|---|
| **30** | cylX protein | Prophage LambdaSa04, terminase, large subunit | Parvulin-like peptidyl-prolyl isomerase |
| **31** | cylD | Hypothetical protein | TnpV |
| **32** | | Portal protein | Tetracycline resistance protein TetO |
| **33** | | Prophage LambdaSa04, ClpP endopeptidase | Hypothetical protein |
| **34** | | Prophage LambdaSa04, HK97 family major capsid protein | ResP |
| **35** | | Phage protein | DNA topoisomerase |
| **36** | | Phage protein | DNA recombinase |
| **37** | | Prophage pi2 protein 37 | Recombinase |
| **38** | | Prophage pi2 protein 38 | DNA recombinase |
| **39** | | Hypothetical protein | Helix-turn-helix domain-containing protein |
| **40** | | Prophage pi2 protein 39 | Nucleotidyltransferase |
| **41** | | Hypothetical protein | Aethyltransferase |
| **42** | | Hypothetical protein | AadE_1 |
| **43** | | RelE | Sat |
| **44** | | Phd_YefM | ISSdy1, transposase OrfA |
| **45** | | Tail protein, phage associated | Transposase |
| **46** | | Hypothetical protein | AphA |
| **47** | | Hypothetical protein | Erythromycin ribosome methylase ErmB |
| **48** | | Hypothetical protein | Hypothetical protein |
| **49** | | Hypothetical protein | Delta protein |
| **50** | | Prophage LambdaSa04, holin | Omega protein |
| **51** | | Cpl-7 lysozyme C-terminal domain | Epsilon-antitoxin |
| **52** | | Putative choloylglycine hydrolase | Toxin of epsilon-zeta postsegregational killing system |
| **53** | | Hypothetical protein | Polynucleotide kinase |
| **54** | | DNA recombinase | AadE_2 |
| **55** | | Resolvase family site-specific recombinase | Toxin of epsilon-zeta postsegregational killing system |
| **56** | | Recombinase | Hypothetical protein |
| **57** | | Site-specific recombinase | Hypothetical protein |
| **58** | | Site-specific recombinase | TraG/TraD family protein |
| **59** | | Erm Leader peptide | Site-specific recombinase |
| **60** | | ErmTR | DNA primase |
| **61** | | Spectinomycin phosphotransferase | Hypothetical protein |
| **62** | | Cytidine deaminase | Glutamate-cysteine ligase |
| **63** | | Cytidine deaminase | ATPases with chaperone activity, ATP-binding subunit |
| **64** | | Zn-dependent hydrolase | Transcriptional regulator |

| | | |
|---|---|---|
| **65** | Group II intron-interrupted relaxase ltrB | Signal recognition particle GTPase |
| **66** | AphA | phosphoesterase |
| **67** | Cytoplasmic protein | ORF10 |
| **68** | TetR family transcriptional regulator | ORF9 |
| **69** | TetR family transcriptional regulator | ORF4 relaxase |
| **70** | Cell wall surface anchor family protein, truncation | NsuP |
| **71** | IS5 family transposase orfB | NsuR |
| **72** | Transposase | NsuK |
| **73** | Transposase | NsuF |
| **74** | HslO | NsuE |
| **75** | Chaperone protein HslO | NsuG |
| **76** | AraC family transcriptional regulator | NsuC |
| **77** | Cell wall surface anchor family protein | NsuI |
| **78** | Cell wall surface anchor family protein | DNA-binding protein |
| **79** | Sortase family protein | Resolvase family site-specific recombinase |
| **80** | Sortase | Orf28 |
| **81** | Cell wall surface anchor family protein | Hypothetical protein |
| **82** | Sortase family protein | Hypothetical protein |
| **83** | Cna B domain-containing protein | lipoprotein |
| **84** | Orf28 | ABC transporter ATP-binding protein |
| **85** | Hypothetical protein | ABC transporter ATP-binding protein |
| **86** | Hypothetical protein | Membrane protein |
| **87** | Lipoprotein | ABC transporter permeae |
| **88** | ABC transporter ATP-binding protein | CylX protein |
| **89** | ABC transporter ATP-binding protein | CylD |
| **90** | Membrane protein | |
| **91** | ABC transporter permeae | |
| **92** | CylX protein | |
| **93** | CylD | |

**Table S6. Primers used for circularization of plasmid pNGBS128.**

| Primer | Sequence | Amplicon Size (bp) |
|---|---|---|
| pNGBS128_F1 | GGGTAAAATTAGGGTTCAGGC | 345 |
| pNGBS128_R1 | TCAGCGGTTACTGGATTATTTG | |
| pNGBS128_F2 | AGCATCCTTACTTTCATGCG | 273 |
| pNGBS128_R2 | ACGGTTAGAAAAAATCGGCG | |
